# Thymoma: An Overview

**DOI:** 10.3390/diagnostics13182982

**Published:** 2023-09-18

**Authors:** Doaa Alqaidy

**Affiliations:** Department of Pathology, King Abdulaziz University, Jeddah 21589, Saudi Arabia; dyalqaidy@kau.edu.sa

**Keywords:** thymus, mediastinum, thymoma, staging, classification

## Abstract

Thymomas are considered one of the most prevalent types of mediastinal epithelial tumors, which frequently develop in the anterior mediastinum. Due to their rarity, these tumors’ nomenclature, classification, and staging are likely to be the subject of debate and argument for most expert pathologists. Furthermore, the significance of thymoma histologic classifications have been debated over the past twenty years. While certain advocates argue that staging at the time of diagnosis is more significant, others believe that histologic subtyping has a significant impact on how patients behave clinically. In this review, we will focus on some of the challenges that diagnostic surgical pathologists may experience while evaluating the histopathology of thymomas and staging these tumors. We will additionally glance over the clinical characteristics of these distinct tumors and the current management strategy.

## 1. Introduction

Thymic epithelial lesions are mostly thymomas. They are a common cause of anterior mediastinal mass in adults [1]. However, in general practice, thymomas account for approximately 0.2–1.5% of all malignant neoplasms. Among the anterior mediastinal tumors, thymoma is the most common. It accounts for 20% of all mediastinal neoplasm in adults [2,3]. Thymomas occur in 0.13–0.26 cases per 100,000 people per year [1,4,5]. They are extremely uncommon in young people and are more prevalent among individuals in their fifth and sixth decades of life. Only 11% of thymomas were diagnosed before the age of 35 [1,6]. It affects females slightly more commonly than males [3,4,7]. In a more detailed analysis of the epidemiology of thymoma, Engels stated in his study that Asian and Pacific Islanders had a greater prevalence of thymomas in the USA [8].

Patients are commonly presented as a single well-circumscribed mass, readily identified by the computed tomography (CT) technique. The majority of thymomas present as round or oval lobulated lesions on magnetic resonance imaging (MRI) scans and computed tomography (CT) [6,9,10]. Areas of calcification and hemorrhage (low densities on CT scan) are rarely seen, although irregularities are often indicators of local invasion and aggressive behavior [1,9,10].

## 2. Clinical Features

Thymoma can present in different ways, including asymptomatically as a mediastinal mass on chest radiography (about one-third of cases), local compressive symptoms as chest pain, a neck mass, or superior vena cava syndrome, or concurrently with myasthenia gravis in one-third of cases [1,7,8].

Although thymoma patients present with a variety of symptomatologies, it is important to emphasize the link between thymomas and paraneoplastic diseases [11,12]. The numerous associations between thymomas and other illnesses, such as autoimmune disorders, collagen vascular disorders, hematological disorders, neoplasia, and others, are well established [1,7,8]. The medical condition that seems to have the strongest association with thymoma is myasthenia gravis. It is widely believed that thymomas are present in roughly 10–15% of myasthenia gravis (MG) patients [13]. Additionally, it has been shown that 20–25% of individuals with MG and 40% of those with thymoma had one or more paraneoplastic autoimmune diseases [2,7,14,15]. In a study by Mao et al. [14,16], the authors stated that out of 2206 potentially relevant studies, the incidence of thymoma in MG patients was 21%. Additionally, thymoma appears to be much more common in male MG patients and those who were older than 40 at the time of MG’s diagnosis [14]. Furthermore, in a study by Okuma et al. [17], immunological abnormalities were found in 21.8% of the thymoma group in a clinicopathological investigation of 187 thymic malignancies, including thymomas, thymic carcinomas, and neuroendocrine carcinomas. In total, 13.3% of these individuals had secondary malignancy [17]. Weissferdt et al., in one of the largest series on thymomas ever reported [18], found that 807 patients out of 1470 patients with thymomas had pertinent clinical data available to them. The authors found the following connections in their examination of 807 patients:

- Myasthenia gravis—17%;
- Neoplasia—6.8%;
- Other autoimmune diseases—3.8%.

In terms of therapy and prognosis, the preferred course of therapy for thymomas is surgical resection, which is typically successful [7,15]. The extent of the tumor’s invasiveness and its resectability, however, are significant factors that are considered when determining if surgical resection is appropriate [19]. The degree of invasion is directly related to the clinical prognosis for patients with thymomas. Complete surgical resection seems to be the preferred course of treatment for individuals whose illness has only spread to the mediastinum and the risk of recurrence is low [20]. On the other hand, invasive disease is more likely to be treated with additional medical techniques, such as chemotherapy and/or radiation therapy [10,16,19]. Even when a full resection is performed, thymoma often recurred, so it is important to plan for a long period of follow-up. Recurrences of thymoma affect between 10 and 29% of individuals. Distant metastasis commonly manifests in the lungs, liver, and bone [7]. The lung is widely recognized as the most frequent location for distant metastases.

## 3. Thymoma Classification

Even though many publications have noted the difficulty in classifying thymomas, specifically due to their heterogeneity and the risk of predicting outcomes based on specific cell types, this is likely one of the most debatable issues in the history of thymomas [2,21]. For classifying thymomas, several histological schemes have been suggested and proposed over the years [2,11,21], Table 1.

Prior to the original 1999 World Health Organization consensus publication on thymoma, the first attempt to classify this heterogenous group of tumors in 1961 was made by Bernatz [22] et al. based on the amount of lymphocytes present in each kind of thymoma into the following categories:

- Lymphocyte-rich thymomas are those tumors where lymphocytes predominate over epithelial cells;
- Epithelial-rich thymomas are those where lymphocytes are present in smaller amounts than the epithelial cells;
- Mixed thymomas, also known as lymphoepithelial thymomas, are those tumors where lymphocytes and epithelial cells are present in roughly equal amounts.

**Figure 1:**
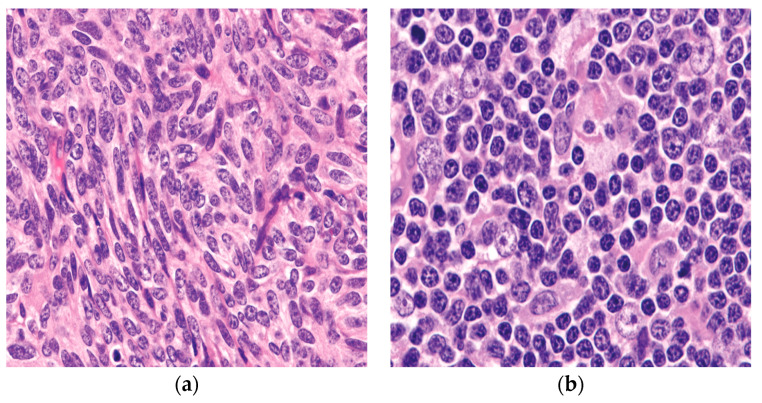
The two epithelial components characteristically seen in thymoma: (**a**) the spindle/oval cells commonly seen in type A and type AB thymoma; (**b**) round/epithelioid epithelial cells found in type B thymoma. (**a**,**b**) (H&E, 40×).

The “histologic-based classification”, which Marino and Muller-Hermelink suggested in 1985 [23], is based on the medulla and cortex of the normal thymus compartment. The tumors are categorized into three categories in this proposal: Tumors that are intended to recapitulate the healthy thymic medulla are known as “medullary thymomas”. However, thymomas that mimic the normal thymic cortex are referred to as cortical thymomas. Finally, mixed thymomas are tumors with medullary and cortical components. Furthermore, a more histologic classification attempt was proposed, and in 1999, because of several differences in how thymomas are classified histologically, the World Health Organization (WHO) proposed an “official” classification system [4,6,21]. The classification depends on the concept that there are two primary histologic kinds of tumor cells in thymomas: round/epithelioid (named type B) and spindle/oval (designated type A) [21], (Figure 1). Type AB was given to tumors that included elements of these two categories. In the current 2021 WHO classification [4], type AB thymoma is also compassing cases of mixtures with a lymphocyte-depleted type A component and a type B-like lymphocyte-rich component. These elements may combine into distinct, independent lobules or may be intermingled. For thymoma type B, three subgroups designated as B1, B2, and B3 were further subclassified on the basis of the proportional increase (in relation to the lymphocytes) and the presence of atypia of the neoplastic epithelial cells [2,4,21]. The former WHO type C thymoma, which was designated to cases that revealed significant cytologic atypia, nuclear pleomorphism, and notable mitotic activity, all of which are signs of malignancy, was removed from the 2004 WHO classification, and since then, these cases are considered thymic carcinoma [2,4,21]. The diagnosis should describe all observable histological types, starting with the main component, and minor components should be estimated in 10% increments for thymomas that have several histological patterns [4].

When discussing thymoma classification, it is crucial to highlight the Suster–Moran proposal which was first introduced in 1999 [24]. According to their proposal, primary thymic epithelial neoplasms are a spectrum of lesions that range from well to poorly differentiated tumors. This assumption served as the foundation for their histologic grading of the tumors [21,24]. Based on the presence or absence of the distinctive organotypical signs of differentiation in the normal thymus and the cytologic atypia, the degree of differentiation for every specific subtype was established as follows:

- Thymoma: Well-differentiated thymic epithelial neoplasms are tumors that exhibit the majority or all the organotypical characteristics of thymic differentiation and lack of cytologic atypia.
- Atypical thymoma: Tumors that exhibit mild to moderate cytologic atypia and only certain organotypical characteristics of thymic differentiation are categorized as moderately differentiated thymic epithelial neoplasms.
- Thymic carcinoma: These tumors are poorly differentiated thymic epithelial neoplasms and are defined by the complete lack of the organotypical characteristics of the thymus and overt cytological signs of malignancy.

It is also vital to note that thymomas are tumors with a complex heterogeneity and many growth patterns, making it challenging to propose a single and simple classification scheme [25,26]. Because of this heterogeneity, any histological schema is rather unrealistic, and the staging of the tumor at the time of diagnosis is more important than ever for determining the best course of treatment [27,28].

### 3.1. Pathological Features

Thymomas can have a wide range of macroscopic characteristics, such as solid, cystic, and areas of infarction/necrosis [27]. The majority of thymomas are well-defined tumors, however, a subset can be ill-defined [1,7,27] with ill-defined capsules. In thymoma cases with prominent cystic or hemorrhagic changes, special attention should be given to proper sampling of the solid areas which usually illustrate the classic thymoma features. Failure to do so will delay the diagnosis and the proper management [1]. Size wise, tumors can range from as tiny as 1 cm to ones with a maximum diameter of far over 10 cm [29]. 

Microscopically, as was already mentioned, there is a broad variety of histological growth patterns that can be observed in thymomas and they can be diagnostic pitfalls. In this section, we are going to summarize the microscopic features of the conventional thymoma. In addition, we will highlight the thymomas’ unusual histological subtype and illustrate how these tumors might be mistaken for other neoplasms and cause diagnostic dilemmas.

#### 3.1.1. Conventional Thymoma with Lymphocytes

**Figure 2:**
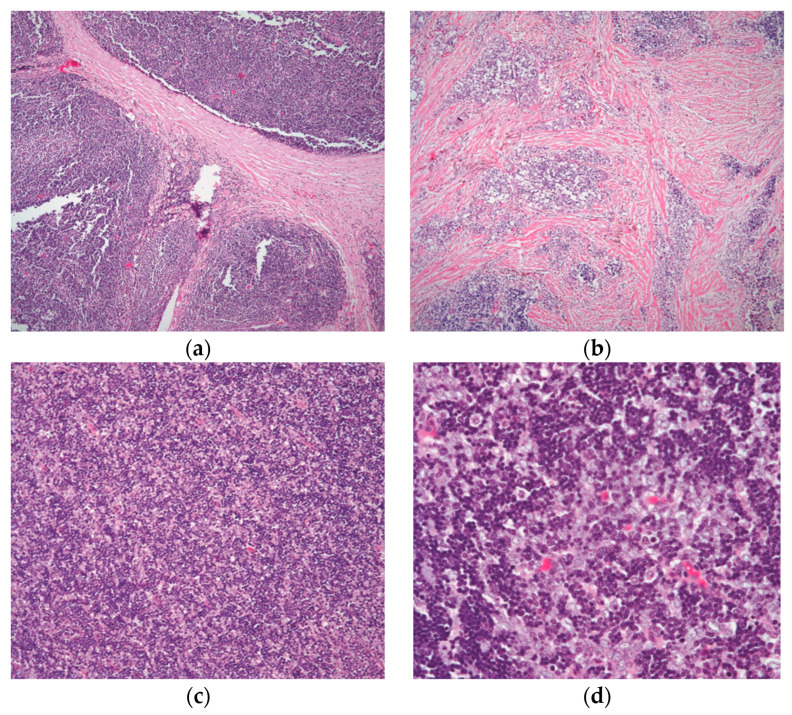
The different growth patterns of conventional thymoma: (**a**) type B1 thymoma showing lobulation by thick fibrous bands; (**b**) thymoma in areas of fibrocollagenous stroma with no well-defined lobulation; (**c**) thymoma with an even distribution of lymphocytes and epithelial cells and a diffuse development pattern; and (**d**) higher magnification displaying the mixed population of lymphocytes and polygonal epithelial cells. (**a**,**b**) (H&E, 4×); (**c**) (H&E, 10×); (**d**) (H&E, 20×).

Included in this category are the tumors that have been classified as mixed thymoma—lymphoepithelial, cortical, and WHO type B2, as well as those that have been identified as lymphocyte-rich, cortical, and WHO type B1 in other nomenclatures [1,21]. Microscopically, this thymoma is lymphocytic rich with minimal epithelial components. It usually has the characteristic perivascular space [7,11] and three distinct growth patterns may be visible in the low-power image of these tumors:

1. A lobulated growth pattern with considerable hyalinization or bands of connective tissue between the lobules (Figure 2a).
2. The tumor lacks well-formed lobules, but there is the presence of collagenous material mixed with the biphasic populations of lymphocytes and epithelial cells (Figure 2b).
3. A diffuse growth pattern in which the tumor exhibits sheets of lymphocytes mixed with epithelial cells in various ratios (Figure 2c,d).

Statistically, these tumors represented 31.1% of the total and 51.9% of the invasive thymoma in that category in a study of 1470 patients that was conducted by Weissferdt et al. [18]. The authors concluded that half of all cases of these types of thymomas may be invasive at the time of diagnosis from the total number of cases of these tumors.

#### 3.1.2. Spindle Cell Thymoma

**Figure 3:**
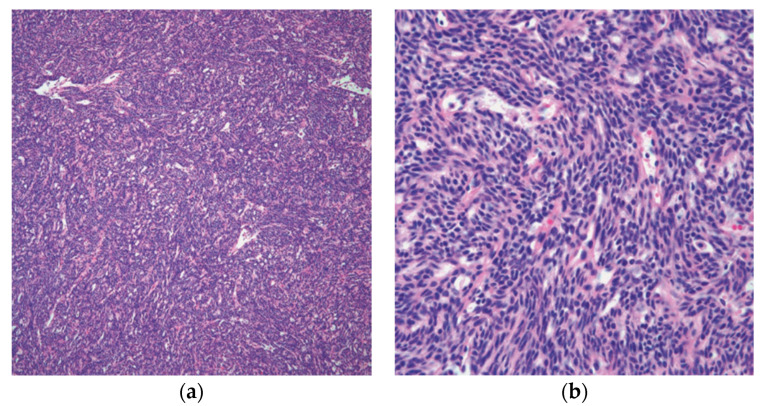
Spindle cell thymoma histologic feature: (**a**) low power of thymoma type A/spindle cell thymoma; (**b**) high power view showing the characteristic spindle cell cytology. (**a**) (H&E, 10×); (**b**) (H&E, 20×).

Spindle cell thymoma has a high degree of morphologic diversity. The tumor cells may be arranged in ways that may resemble other epithelial or mesenchymal neoplasms, but their fundamental morphology is that of a spindle cellular proliferation made up of fusiform cells with sparse cytoplasm, elongated nuclei, dispersed chromatin, and inconspicuous nucleoli [19,25] (Figure 3).

These tumors can exhibit three distinct growth patterns at low power magnification: lobulated, diffuse, and vascular or HPC growth pattern [30,31]. Additionally, regions of hyalinization, which can range from focal to widespread, may be seen in spindle cell thymomas. The lymphocyte component can vary in this thymoma from none or few lymphocytes to prominent lymphocytes intermixed with the spindle cells. In the WHO classification, this thymoma is classified as type A or type AB. From a statistical perspective, spindle cell thymomas make up around 13% of all thymomas [31,32].

#### 3.1.3. Atypical Thymoma, WHO Type B3 Thymoma

**Figure 4:**
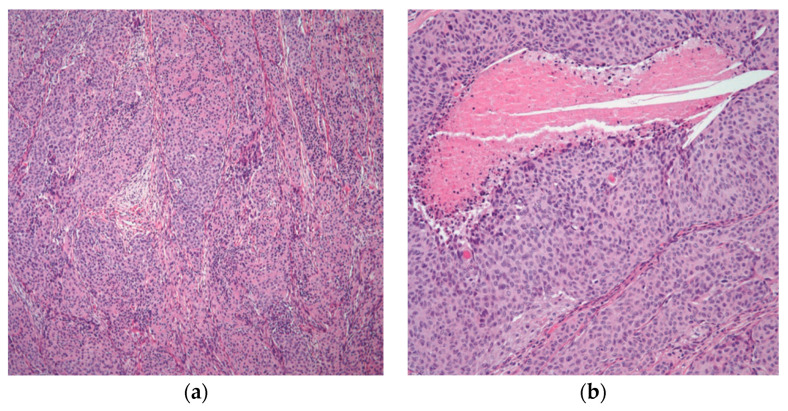
Atypical thymoma/WHO type B3: (**a**) atypical thymoma case showing nested pattern and rosette-like formation; (**b**) higher magnification showing atypia and focal comedo necrosis. (**a**) (H&E, 4×); (**b**) (H&E, 10×).

This entity has histological characteristics and clinical behavior that are more similar to thymomas than to carcinomas, and as a result, the term “atypical thymoma” is best used to describe an entity with characteristics that fall somewhere between thymomas and thymic carcinomas [2,24,33]. Morphologically, the epithelial proliferation in this specific type of thymoma is characterized by round to polygonal cells with an abundance of eosinophilic cytoplasm, vesicular nuclei, several cells with conspicuous nucleoli, and occasionally mitotic figures [27,34]. More significantly, these tumors have a sparse population of T-immature lymphocytes, which is essential (Figure 4).

Atypical thymoma (WHO category B3) appears to be more frequently attributed to invasion, more rapid recurrence, and a higher risk of tumor-related fatalities than the better-differentiated forms of the disease [2,21,35]. In a large cohort study by Weissferdt et al. [18], they examined 1470 thymomas, of which 186 were atypical thymomas, accounting for around 12.7% of the total. In total, 139 of the 186 atypical thymomas in this cohort were invasive tumors at various clinical stages, roughly 75% of the cases. As a result, the distinction between thymomas and atypical thymomas became statistically significant when a survival curve was analyzed.

### 3.2. Unusual Histologic Subtypes

#### 3.2.1. Micronodular Thymoma with B-Cell Lymphoid Hyperplasia

**Figure 5:**
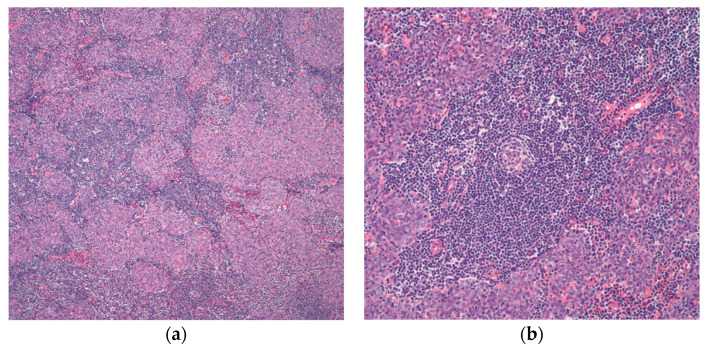
Micronodular thymoma with B-cell lymphoid hyperplasia: (**a**) islets of nodular epithelium embedded within a lymphocytic stroma; (**b**) many germinal centers are easily identifiable. (**a**) (H&E, 2×); (**b**) (H&E, 10×).

The first description of this specific type of thymoma was by Suster and Moran in 1999 [36], which is characterized by the presence of lymphoid nodules that have significant germinal centers and spindle cell nodules. The oval cells that make up the spindle cell nodules lack nuclear atypia and mitotic activity (Figure 5).

Cystic changes can be prominent in this type of thymoma. Oramas et al. reported the clinopathological features of 25 cases of micronodular thymomas with prominent cystic changes. In this case series, four cases were invasive tumors with invasion into the perithymic adipose tissue through the fibrous capsule. However, the majority of them (21 tumors) were encapsulated [37]. By immunohistochemistry, the epithelial spindle cells are positive for cytokeratin, while the lymphoid component was shown to be mainly B-lymphocytes [1,37]. In the clinical follow-up provided in this initial publication [36], as well as in the subsequent studies, the patients were free of recurrence in around half of the cases. This clinical behavior was like that seen in conventional thymomas.

#### 3.2.2. Other Histologic Variants

Thymoma with significant plasma cells, also known as plasma cell-rich thymoma, is one of the rare histologic types that appears to frequently be associated with an underlying autoimmune illness, particularly myasthenia gravis, [27,38]. It shows a mixture of both epithelial and plasma cells. It is also important to note that plasma cells are one of the normal cellular components of the normal thymus, and they may be present in the normal thymus, although not in the same proportion as lymphocytes [3,7,39]. Metaplastic thymoma is another unusual histological type of thymoma. It is characterized by a spindle cell component mimicking sarcoma with minimal lymphoid component. This specific variant has been described in 1997 under the term of thymoma with pseudosarcomatous component [40]. Other rare variants of thymomas can have myoid cells, showing papillary/pseudopapillary, adenomatoid-like, alveolar, glandular, signet ring cell, and clear cell features. It is important to bring attention to the difficulty that these thymoma variations may present in tiny mediastinoscopic biopsies, potentially leading to an incorrect diagnosis. Those tumors, nonetheless, need to be treated appropriately under the term “thymoma”, and more crucially, they do belong to certain categories rather than a specific position in any classification scheme [27].

## 4. Immunohistochemical and Molecular Characteristics

Although thymoma is often diagnosed morphologically, various investigations have provided some insight into the immunohistochemical characteristics of these tumors. There is currently no established unique immunohistochemical marker for malignancies originating from thymic epithelial cells [41]. Generally, the epithelial component is usually positive for broad-spectrum cytokeratin, cytokeratin 5/6, while CD45 and TDT usually highlight the T-cell component. Occasionally, the epithelial cells can express polyclonal PAX8, calretinin, TTF-1, CD5, CD117, synaptophysin, CD56, and PD-L1 [41,42], and they are generally negative for monoclonal PAX8 and EMA. 

Several studies have investigated the role of high expression of programmed cell death 1 ligand (PD-L1) in thymic epithelial neoplasm, including thymoma [42,43]. Wei et al., in their cohort, examined the PDL1 expression in 100 surgically treated thymomas and they stated that high PD-L1 expression was associated with advanced Masaoka staging and with high-grade histology [43]. In a similar study by Weissferdt et al. [42], expression of PD-1 was detected in 46/74 thymomas (62%). In their cohort, neither PD-1 nor PD-L1 expression appeared to be related to patient outcomes, which raises questions about the usefulness of these markers as prognostic indicators. However, neoadjuvant treatment was linked to PD-L1+ cases in thymoma. Also, this study illustrated that up to 82% of thymic epithelial neoplasms express PD-1 and/or PD-L1. These findings support the need for PD-1/PD-L1 targeted treatment for these malignancies, but their prognostic predictive value is yet unknown. 

In the past decade, many investigations have been conducted in an effort to characterize the molecular profile of thymic epithelial neoplasms, including thymoma. Thymomas typically have low mutational burdens and a variety of copy number abnormalities [44,45] (summarized in Table 2). The observed low mutation burden in thymomas has been associated with the smaller number of somatic mutations present in the DNA of neoplastic cells [46].

The most common molecular alteration in thymoma type A, type AB, type B2, and type B3 is the loss of heterozygosity across chromosome 6 (the 6q25.2-p25.3 region contains the FOXC1 tumor suppressor gene) [45,47]. This particular alteration was not detected in thymoma type B1. In addition, missense mutations in GTF2I p.L424H are the most frequent recurring genetic change, mainly in thymomas type A and type AB (occurring in up to 38% of cases). It is linked to a lower prevalence of myasthenia gravis and a better prognosis. Researchers observed a pattern of GTF2I and BCOR mutations that are mutually exclusive in thymomas from individuals with myasthenia gravis [46]. While NRAS and TP53 mutations are substantially more prevalent in type B2 and B3 thymomas and thymic carcinomas, mutations in the HRAS gene are primarily restricted to type A and AB thymomas [48,49]. Finally, thymomas have not been found to have any targetable mutations, such as those in the EGFR or KIT genes [45,50]. For patients with invasive diseases that cannot be treated by surgery alone, more research is required in order to shed some light and provide information regarding helpful targeted therapies [44,47].

## 5. Thymoma Staging

In thymoma, staging is the most critical step in patient management as it has been linked to prognosis. Many staging system schemes have been published in the literature throughout the years. In this section, we will give a historical summary of how these tumors have been staged, with a focus on current developments in this area (summarized in Table 3).

One of the earlier staging systems was presented in 1981 by a Japanese team led by Masaoka [51]. This system put a strong emphasis on the severity of the disease, and it became the cornerstone for the subsequent schemes. The thymoma was divided into the following four stages based on macroscopic and microscopic evaluation:

- Stage I: macroscopically fully encapsulated with no capsular invasion.
- Stage II: macroscopic invasion into surrounding fatty tissue or mediastinal pleura or microscopic invasion into the capsule.
- Stage III: macroscopic invasion into the surrounding organs, such as the pericardium, great vessels, or lung.
- Stage IV: (a) pleural or pericardial dissemination or (b) lymphoid or hematogenous metastasis.

In the subsequent generation of Masaoka staging systems, updated by Koga, the Koga-modified Masaoka staging system has been the most frequently adopted to stage thymic epithelial neoplasms [52]. It is noted accurately in this amended staging system that a tumor cannot be categorized as invasive if it does not penetrate through the capsule [53,54,55]. The modified Masaoka stage II tumors have either microscopic trans-capsular invasion or macroscopic invasion into the perithymic fatty tissue. Cases adhering to the pericardium or mediastinal pleura but not rupturing them are also considered stage II.

More recent and pathologically user-friendly schemas for staging thymoma and thymic carcinoma have been presented, taking into account the fact that the mode of spread for these tumors is frequently divergent [53,56,57]. Moran et al.’s subsequent staging system, based on over 200 cases of thymoma, was designed with the goal of informing the clinical team of the precise anatomical regions that the tumor cells had penetrated. This is helpful in distinguishing thymomas that are localized to the mediastinum from those that are infiltrating nearby anatomical tissues [34]. This could spare patients who would benefit from full surgical resection alone from receiving extra therapy. It is also vital to note that a more tailored approach to treatment may be used if particular information regarding the anatomy that is involved is provided [54]. This staging system, as illustrated in Table 2, gives a precise description of encapsulated versus invasive thymomas, as well as definitions of the phrases “pleural” and “pericardial involvement”, respectively. The term “drop metastasis” is also discussed and is defined as discontinuous tumor extension to the diaphragm or other intrathoracic structures (stage IIIa) as opposed to direct tumor infiltration into intrathoracic structures (stage II) or lymph node metastasis or hematogenous extrathoracic spread (stage IIIb). Additionally, the idea of stage 0 is presented to highlight how similar these tumors are to in situ tumors at other organ locations, where total removal of the lesion would probably lead to a complete cure.

Lately, the American Joint Committee on Cancer (AJCC) [58] has updated its guidelines to include the new TNM proposal [59,60], which T is not based on the tumor size. However, it is related to the tumor capsule integrity and tumor invasion into the adjacent structure (Table 2). The concept of adopting a TNM staging system for thymoma started a long time ago when Yamakawa et al. [61] presented a series of 207 cases of thymomas, in one of the early attempts to develop a TNM staging system for thymomas, and noted that “only a few cases had lymph node metastases”, adding that the TNM system has a minor advantage over the traditional staging systems. Additionally, it can be more suitable for thymic carcinoma than thymoma. 

It is clear how significant appropriate staging plays in predicting the clinical course of thymomas. Regardless of whether Masaoka and Moran’s proposals or the TNM staging is used, the staging must be reliable and useful for clinical use. 

## 6. Management and Treatment Strategies

Surgical excision has been widely accepted as the preferred and most effective treatment modality for thymomas, regardless of their specific histological classification. This is evidenced by the high overall and disease-free survival rates it achieves in patients with stage I and stage II disease [7,20]. The conventional procedure involves performing a median sternotomy, followed by a complete thymectomy and excision of the tumor. The 5-year survival rates following surgical removal of stage I thymoma vary between 80% and 100%, with an average rate of 91%. In cases of more invasive lesions, particularly those classified as Masaoka stage III, where the tumor has invaded the surrounding tissues, the preferred therapy remains definitive surgical removal. 

It is important to acknowledge that a more extensive surgical procedure may be necessary (including possible resection of part of the pericardium and a portion of the lung) [35,62]. In cases when the hemidiaphragm is involved, it may also be subject to resection and subsequent reconstruction with a prosthetic patch, often composed of polytetrafluoroethylene (PTFE). The primary objective in managing these lesions is to achieve a complete resection, preferably with clear margins. Thymoma resection can be performed using a variety of methods. The most common method is a median sternotomy. Several minimally invasive techniques have been documented in the literature, such as transcervical, extended transcervical or video-assisted thoracoscopic, and robotic surgery. These techniques involve various approaches, including right, left, cervical, and sub-xiphoid approaches [63].

Thymic neoplasms have a notable sensitivity to chemotherapy, resulting in a response rate that varies between 30% and 60% in patients with locally advanced diseases [20,63]. Chemotherapy may, thus, have a significant impact as a neoadjuvant intervention for those who are not eligible for surgery, as well as serving as an adjuvant therapy to enhance the long-term, disease-free survival rate for patients who have already had surgical procedures. In general, cisplatin-based chemotherapy regimens are safe and effective [7,64].

Furthermore, invasive thymomas respond to radiation therapy, making it a potential adjuvant therapy for improving local tumor control and survival. Although its efficacy in stage II patients is still up for debate, postoperative radiation has been found to reduce the recurrence rate of thymomas by 50–20% in stages III and IV [65]. Radiation therapy, both before and after surgery, has not been shown effective for treating advanced illness [66]. 

Finally, it has been generally accepted that the best treatment for thymoma recurrences and advanced thymomas is multimodal treatments that combine radiation and chemotherapy with surgery, where feasible. Some studies have found that an extra surgical excision of pleural implants improves oncological outcomes. Pleuropneumonectomy has been replaced by partial or total pleurectomy/decortication due to its high morbidity and mortality rate. Intracavitary perfusion of chemotherapeutic agents, hyperthermic intrathoracic chemotherapy (HITHOC), after surgical local excision of recurrences is one of the modalities used for recurrence pleural implants. This approach has been shown to be safe, feasible, and effective, as evidenced by the favorable survival rate and enhanced management of local disease. In a retrospective research conducted by Aprile et al. [67], the authors assess the oncological efficacy of hyperthermic intraperitoneal chemotherapy (HITHOC), specifically focusing on its ability to control local disease. The study compares a group of 27 patients who had HITHOC with a group of patients who did not receive this treatment. They concluded that this therapy method may be regarded as a valuable tool for achieving local disease management and demonstrates both safety and technical feasibility when administered to carefully selected patients with a longer local disease-free interval compared to surgery alone [67].

## 7. Conclusions

It is critical to emphasize that most general pathologists still struggle with the diagnosis and categorization of thymomas. A detailed clinical and radiological evaluation is necessary for the diagnosis. The most crucial issue is to be aware of the many patterns of growth that thymomas might exhibit in order to prevent misdiagnosis. Even though the TNM staging system is a useful method for thymoma staging, many studies view the modified Masaoka system as the gold standard. Proper anatomical staging continues to be of significant assistance to the clinical team in directing their therapy.

## Figures and Tables

**Table 1:** Different histological classification systems.

Bernatz	Muller-Hermelink	Moran–Suster	WHO, 6th Edition
Spindle cell thymomas Mixed thymomas Lymphocytes-rich thymomas	Medullary thymoma Mixed thymoma Predominantly cortical Cortical thymoma	Thymoma Thymoma Thymoma Thymoma	Type A Type AB Type B1 Type B2
Epithelial-rich thymomas with cytologic atypia	Well-differentiated thymic carcinoma	Atypical thymoma	Type B3
	Thymic carcinoma	Thymic carcinoma	Thymic carcinoma

**Table 2:** Common mutations in different histologic subtypes of thymoma.

Molecular Alteration	Thymoma Histologic Subtypes
Loss of heterozygosity across chromosome 6 (FOXC1 6q25.2-p25.3)	Common in thymoma type A, type AB, type B2, and type B3
Missense mutations in in GTF2I p.L424H	Mainly in thymomas type A and type AB (38% of cases)
HRAS gene mutation	Restricted to type A and AB thymomas
NRAS and TP53 gene mutations	Common in type B2 and B3 thymomas and thymic carcinomas
GTF2I and BCOR mutations	Mutually exclusive in thymomas from individuals with Myasthenia Gravis

**Table 3:** Different staging systems for thymoma.

TNM	Definition	Mazaoka/Koga	Moran
TX	Primary tumor cannot be assessed	NA	NA
T0	No evidence of primary tumor Encapsulated tumor	NA	Encapsulated thymoma
T1	Encapsulated tumor T1a- No mediastinal pleural involvement T1b- Direct invasion of mediastinal pleura	Stage I NA Stag II	Minimally invasive thymoma without involvement of any adjacent structure
T2	Direct invasion of pericardium	Stage III	Stage IIA (innominate vein, mediastinal pleura, lung) Stage IIB (pericardium)
T3	Invasion into lungs, brachiocephalic vein, superior vena cava, phrenic nerve, chest wall, extrapericardial pulmonary artery or veins	Stage III	
T4	Invasion into aorta, arch vessels, pulmonary artery, myocardium, trachea, esophagus	Stage III	Stage IIC (great vessels and heart)
M1a, b	M1a: The tumor has spread to the lining of the lung, called the pleura, or the lining of the heart, called the pericardium M1b: The tumor may have spread to the lining of the lung or the heart	Stage IVa: pleural, pericardial dissemination Stage IVb: lymphogenous Hematogenous	Stage III—metastatic disease IIIA—intrathoracic structure Diaphragm (drop metastasis) Lymph nodes IIIB—extrathoracic

NA: not applicable.

## Data Availability

Not applicable.
